# A qualitative study of e-cigarette use among young people in Ireland: Incentives, disincentives, and putative cessation

**DOI:** 10.1371/journal.pone.0244203

**Published:** 2020-12-28

**Authors:** Joan Hanafin, Luke Clancy

**Affiliations:** TobaccoFree Research Institute Ireland (TFRI), FOCAS Institute, TU Dublin, Dublin, Ireland; University of Calfornia San Francisco, UNITED STATES

## Abstract

**Background:**

Smoking prevalence in Ireland is falling in all age groups, but e-cigarette use is rising among young people. This qualitative study explores young people’s accounts of e-cigarette use in Ireland.

**Methods:**

Semi-structured individual (22) and focus group (8) interviews were conducted with 62 young people aged 18–22 years, recruited from a higher-education institution and youth organisations working with early school-leavers across Dublin. All were smokers or ex-smokers; 41 had tried e-cigarettes, 11 continued as dual users. We identified themes using thematic data analysis.

**Results:**

Three broad themes were identified: incentivising features, disincentivising features, and ambivalent and unsuccessful cessation, named putative smoking cessation. Incentivising features included price, pleasing taste/ flavours, and the possibility of indoor use. Disincentivising features related to adverse health effects (pain, discomfort, sore throat, coughing, headache) and unpleasant physical effects (bad taste, problems resulting from device faults). Other disincentives were over-consumption arising from inability to control intake, "greater addictiveness", product taste, and device faults. Putative cessation refers to the conflict between participants' expected use of e-cigarettes as a smoking cessation/reduction aid and their observed reality of e-cigarettes’ failure in this regard, with reported outcomes including: failure to quit or reduce; continued or resumed cigarette and/or roll-your-own smoking; dual use of e-cigarettes and other tobacco products; and inability to quit e-cigarettes.

**Conclusions:**

Participants were sceptical about e-cigarettes’ "purported relative healthiness", concerned about addictiveness and potential long-term health consequences, and critically aware of advertising and industry strategies. E-cigarettes were viewed as being less denormalised, in part because they could be used in indoor spaces where smoking is banned in Ireland. Although price, taste, and perceived renormalisation were important motivators for young people's use of e-cigarettes, they wanted to quit smoking. The regulation of e-cigarettes through age restriction of access, licensing of outlets, pricing, point of sale and advertising restrictions as well as through the banning of indoor use should be considered by legislators and tobacco control policymakers.

## Introduction

Ireland has a wide range of legislative measures and health promotion policies [1] to reduce the numbers of people smoking (S1 Appendix). Smoking prevalence in the general population has been falling steadily from 31% in 1998 [2] to 17.0% in 2019 [3], and among 16 year olds from 41% in 1995 to 14.4% in 2019 [4]. However, levels of Electronic cigarette (e-cigarette) “last 30 day use” among 16 year olds continue to increase, from 3.2% in 2014 [5] to 10.1% in 2015 [6] to 15.4% in 2019 [4].

E-cigarettes, commercially developed in 2003 as an alternate nicotine delivery device for tobacco smokers [7], are "products that deliver a nicotine-containing aerosol (commonly called vapor) to users by heating a solution typically made up of propylene glycol or glycerol (glycerine), nicotine, and flavoring agents” [8]. By 2013, the major multinational tobacco companies had entered the e-cigarette market [8]. E-cigarettes are marketed via television, the Internet, and print advertisements (often featuring celebrities) [9] as healthier alternatives to tobacco smoking, as useful for quitting smoking and reducing cigarette consumption, and as a way to circumvent smoke-free laws by enabling users to “smoke anywhere” [10]. E-cigarette companies have rapidly expanded, using aggressive marketing messages similar to those used to promote cigarettes in the 1950s and 1960s [10–13]. Young people have long been recognised as a crucial market for tobacco products [11] and e-cigarette marketing strategies that focus on young people ensure that e-cigarette products appeal specifically to them by integrating them into their lifestyle [11], using mass media campaigns [14], and particularly new (social) media campaigns that offer “high reach among audiences no longer accessible via traditional media” [11].

These strategies have proven successful, with known motivations for use of e-cigarettes among young adults including ability to use in smoke-free areas [15] and being perceived as less harmful than combustible cigarettes [15–17], and perhaps even “good’ for them [15]. Perceptions of safety potentially reflect e-cigarette marketing, particularly (inaccurate) descriptions of aerosol as ‘water vapor’ [15]. Other drivers include experimentation, especially within the context of the culture of technology [15, 16]; curiosity [17]; price [14, 15, 18]; and flavours, especially sweeter ones [15], and taste [16].

Much of the discussion of e-cigarettes among health authorities has concentrated on the product itself, its potential toxicity, and use of e-cigarettes to help people quit smoking [7, 8]. Potential negative consequences of e-cigarette use that have been identified include a series of risks from nicotine for the developing adolescent brain [18]; that e-cigarette use may negatively impact cardiovascular health [19]; and flavorants in e-cigarettes presenting potential hazards such as obstructive lung disease [20]. There is evidence that e-cigarettes are part of an emerging pattern of health-risk behaviours for some young people, with e-cigarette users having a higher risk of adolescent adjustment problems, delinquent behaviour, and substance use relative to nonusers [21]. Lack of knowledge about e-cigarettes has been noted, with one study of perceived threats and barriers to e-cigarette use during pregnancy, identifying perceptions of nicotine-related health risks and potential risks of ingredients, and barriers to use including lack of satisfaction and social stigma [22].

Less is known about young adults’ understandings of negative consequences of e-cigarette use. Regarding risks associated with e-cigarette consumption, adolescents have been found to lack information about the dangers of e-cigarette use, being readily able to describe negative consequences of cigarette use but being much less sure regarding risks of e-cigarette use [14, 18]. Conversely, they were found to describe few benefits of cigarettes but a number of benefits of e-cigarette use [18]. The absence of information about the relative harms of e-cigarettes may lead young people to form positive opinions about them [14, 18].

Considerable uncertainty about the constituents and safety of e-cigarettes [23] has been identified as well as ambiguity regarding e-cigarettes’ status and efficacy, and their physical and social risks [24]. Rooke and colleagues identified a key cross-cutting concern among young adults in their study that centred on the safety and health effects of e-cigarettes, for example, whether nicotine was harmful or what was inside the product, and also the potential of e-cigarettes to threaten smoking cessation, identifying them as a “slippery slope” [23]. Also, the perception that e-cigarettes are capable of delivering large doses of nicotine, in shorter consumption sessions [16] has been found to be associated with concerns about inability to control nicotine intake and consequent excessive nicotine consumption [15].

The link between e-cigarette use in adolescents and increased smoking is widely accepted and supported by several reviews [25–27]. The nature of the link and especially causality needs further clarification. At present the gateway theory [28], the common liability theory [29] and the catalyst model [30] are most widely considered but require further examination. The role of e-cigarettes in smoking cessation as well as dual use, particularly for young people, has been a key topic.

Dual use among adults is not homogenously practiced. Robertson and colleagues found that practices developed in multiple ways, including as a means of managing perceived “inauthenticity” of e-cigarette use compared with smoking; through rationalising decreased tobacco use rather than smoking cessation as “success”; as a way to reduce the financial burden of smoking and to circumvent smoke-free policies; and as an attempt to comply with social group norms and manage stigma [31]. A scientific review of 82 studies on e-cigarettes concluded that "the evidence available at this time, although limited, points to high levels of dual use of e-cigarettes with conventional cigarettes, no proven cessation benefits, and rapidly increasing youth initiation with e-cigarettes" [8]. The review further notes that although some cite a desire to quit smoking by using e-cigarettes, other common reasons for using the products are "to circumvent smoke-free laws and to cut down on conventional cigarettes, which may reinforce dual use patterns and delay or deter quitting" [8]. Nonetheless, young people continue to cite the desire to quit smoking as a motivation for e-cigarette use [15], citing the influence of advertising campaigns that describe e-cigarettes as a possible means for quitting conventional cigarettes [18].

In this study, we report our findings from a qualitative study of e-cigarette use among adolescent and young adult smokers in Dublin, Ireland. Our earlier survey had estimated the prevalence, and documented the features, of e-cigarette usage in Irish teenagers [5]. Noting the steep increase in prevalence and the trajectory of adolescent usage internationally and in the European Union [32] we set out to gain a deeper understanding of the nature of young people’s use of e-cigarettes. In this way it was hoped to contribute to the knowledge base that will help Ireland attain its Tobacco Free Ireland status by 2025. To help us do that we explored the nature of young people’s e-cigarette use including:

a) Initiation experience, b) Motivations for use, c) Current patterns of use, d) Feelings associated with the product, and e) E-cigarettes usage in smoking cessation. Our main aim was to inform policy surrounding e-cigarettes legislation and regulation in Ireland in order to reduce young people’s use of nicotine and the possible disruption of the decline in smoking which had been particularly evident from 1995 to 2015 [33].

## Methods

### Approach and rationale

A qualitative approach [34] was adopted in this study in order to explore the reasons behind the growing levels of e-cigarette use in Ireland. An interpretive paradigm underpins this qualitative study of young people and e-cigarettes. Central to this paradigm is understanding how others interpret and make sense of their world [35], sharing the social constructionist goal of “understanding the complex world of lived experience from the point of view of those who live it” [36]. Because of individuals' uniqueness, there are multiple interpretations of the same phenomenon and qualitative research involves “presenting or interpreting people's views, interactions or values” [37]. Therefore, multiple realities were constructed [38] and multiple perspectives presented [39], in this case, those of young women and men in higher education and further education contexts.

This study was conceived following previous cross-sectional survey research that showed noteworthy and increasing levels of e-cigarette use among Irish adolescents, including among non-smokers [5]. As qualitative research prioritises process rather than outcome and allows for an emphasis on meaning [40], it was deemed an appropriate approach for this study as it allowed for the generation of understanding about a specific life experience, namely how young people deal with, and make sense of, e-cigarette use.

### Sample and recruitment

Purposive sampling, a non-probability sampling approach [41], was used. Participants in the study were aged between 18 and 22 years and comprised two groups. One group (three centres, 34 students) was recruited through a youth organisation (Youthreach) which works with early-school-leavers (ESL) from disadvantaged areas. The other group (two centres, 28 students) comprised students (HES) attending a higher-education institution in Dublin city centre.

Eight focus group interviews (40 participants, 4 all-male and 4 all-female groups) and 22 individual interviews were conducted. The individual and focus group interviews with the higher-education students were conducted in classrooms within these institutions (two sites). The interviews and focus groups with early school-leavers took place in the premises of youth organisations across Dublin (three sites).

### Data collection

Individual interviews are often considered the undisputed ‘gold standard’ of qualitative data collection methods, while focus groups are increasingly favoured to explore participants’ experiences in an interactive format [42]. Many researchers favour combining these two methods [43], including in qualitative research on e-cigarettes [15, 23]. Combining methods can threaten trustworthiness if done in an ad hoc way [43], therefore our combined approach was purposeful. Specifically, as qualitative research uses multiple methods or data sources to develop a comprehensive understanding of phenomena [44], we used both individual interviews and focus group interviews as a further means of triangulation. While individual interviews can elicit rich information about personal experiences and perspectives, focus groups elicit data from a group of participants who can hear each other’s responses and provide additional comments that they might not have made individually [45.]. The identification and sharing of various perspectives on the same topic through participant interaction in focus groups is central to their success [45]. Data source triangulation using individual interviews and focus groups has been found to allow data to be explored more deeply, with the combined data leading to an enhanced understanding of the context of the phenomena, and convergence of the data leading to enhanced trustworthiness of findings [42].

Individual and focus group interviews elicited participants’ views about e-cigarettes, using a prepared guide which contained questions focusing on participants’ initiation experiences, awareness, access, usage, positive and negative feelings towards e-cigarette products, and user patterns. Specific probes were used about cost, packaging, and accessibility. Participants were also questioned about smoking of pre-manufactured cigarettes (PMC) and about use of roll-your-own cigarette (RYO) use. This paper presents the data about e-cigarette use and, as participants sometimes made comparisons, about PMC/RYO use as it relates to e-cigarette use.

Questions focused on learning about the behaviours and opinions of young people towards e-cigarette products. In particular, the questions centred on understanding the experiences of ‘why’, and ‘how’ young people use these products; ‘what’ they like or dislike about the products, with ‘whom’ they use them, and ‘where’ they use them. The individual and focus group interview guides were created to give flexibility to the participants so that their responses could be probed and explored fully. Individual interviews lasted from 15 to 45 minutes, median 34 minutes, with focus group interviews running from 50–63 minutes, median 53 minutes. A copy of the individual and focus group interview guides can be found in (S2 Appendix).

### Ethics

Ethical approval was received from the Dublin Institute of Technology’s (DIT) (now Technological University Dublin [TUD]) Research Ethics Committee. Informed consent was given by all participants and participants were assured of confidentiality. In keeping with that, only pseudonyms were used. No participant’s real name was used at any stage as real names were replaced by pseudonyms in the transcripts and only these pseudonyms were used during data analysis and drafting of the paper. We used pseudonyms to allow the reader to track and differentiate the quotes across different participant responses. Additionally, the use of pseudonyms “humanises” participants which we believe is important. while offering confidentiality, and not compromising participants’ identity. In reporting participants’ (pseudonymous) quotes, we also use participants’ gender (M/F) and educational affiliations, higher-education students (HES) or early school-leavers (ESL).

### Data analysis

The data were formally analysed using an iterative, sequential process [39] which involved organisation, immersion, and the generation of categories and themes through coding. This thematic analysis approach [46] commenced with the recordings from the interviews being transcribed verbatim. The analysis of each data set began with each transcript being read in its entirety before notes were made outlining any interesting comments or findings. The transcripts were uploaded to NVivo 11 and preliminary codes generated. Using an inductive analytic approach, low-level, descriptive codes that resulted from the NVivo coding and the annotations from the transcripts were gathered together to form conceptual categories (Categoric analysis). From there, a number of interpretive themes and sub-themes were generated (Theoretical analysis). These themes and sub-themes framed the overall analytic structure and shaped the presentation of findings (S3 Appendix). Themes and sub-themes were reviewed separately and together by the authors, and refined and subsequently compared and contrasted.

### Trustworthiness

The research process was conducted consistently in order to ensure reliability and trustworthiness, and verbatim interview transcripts were peer-checked by the authors as a means of triangulation. Since the main goal of interpretivism is to develop a contextual understanding of unique cases with limited numbers, it is not possible to generalise findings [37]. In presenting our findings, we indicate frequency of occurrence of specific themes to make statements such as “some”, “usually”, and “most” more precise [47]. Sometimes considered controversial [47], this “quasi-statistical analysis style” is one of four major analysis styles used by the wide range of qualitative researchers [35, 48]. More recently, however, Fife (2020) has made a case for adding counting as a standard component for all qualitative data analysis. We use counts here as an addition to standard ways (reflexivity, triangulation, saturation) of checking reliability of our qualitative data. As noted, we do not seek to generalise the findings but rather, as Fife suggests, blend counting with more traditional qualitative presentation of our interview material in order to discover patterns in the evidence, and to allow for “both the easily seen and the virtually invisible to come under examination” [49].

## Results

S1 Table shows demographic characteristics, data collection methods, and smoking and e-cigarette histories for the 44 participants aged 18–22 years whose data are presented below and in S3 Appendix. Of these, 41 were current or previous users of e-cigarettes, with 11 being current dual users. All of the participants were either smokers or ex-smokers. We indicate participants’ gender, and whether they were early-school-leavers (ESL) from disadvantaged areas or more advantaged higher-education students (HES). We observe that, compared with higher-education students, early school-leavers show a strong tendency to have started smoking (combustible cigarettes) at an earlier age (S1 Table).

### Access and context

As regards access and purchase of e-cigarettes, participants accessed them from parents and friends, and purchased them from retail outlets such as supermarkets and corner shops, with no difficulties in accessing e-cigarettes reported: *They’re pretty much everywhere now*, *so when first bought it*, *I just assumed that it would be there (in her local shop) and I went there and got it* [Niamh, F, ESL].

Two participants mentioned ease of access as a positive feature of e-cigarette use. Parents had purchased e-cigarettes for a small number of students (n = 4).

Students demonstrated a critical consciousness about e-cigarette devices and were aware of advertising strategies, including the use of celebrities, exotic travel, competitions, and “sponsored vapists”:

P7: *Yeah it’s more stylish you know*? *It’s like an accessory*.
P5: *Yeah*, *Leonardo Di Caprio was puffing on one recently*.
P2: *It’s a fad though*.
P7: *You’ve seen those ads like on tv and stuff*, *advertising e-cigarettes and they’re all stylish you know*, *like someone driving a car in the desert*, *and pulls on an e-cigarette*, *and it’s like ‘when you’re in the desert…’* [FG, M, HES].

and again:

P3: *Yeah*, *like one of my mates is a sponsored vapist*. *I swear to god*, *he’s paid by a vape shop in competitions now*. *In [name of shop] in town*, *it’s actually like ridiculous but [name of shop] have vape competitions* [FG, M, HES].

Students were also mindful of the influence of the tobacco industry:

P5: *Yeah*, *and even when you do hear that they’re bad*, *you can’t be sure that it’s not tobacco companies lobbying to get…*
P1: *Yeah*, *like at the end of the day*, *they’re all bad*. *Like you’re dosing yourself in addictive substances*.
P4: *Yeah*, *like you shouldn’t be doing that to your lungs* [FG, M, HES].

Three substantive themes emerged from the data analysis, *viz*.: features of e-cigarettes that incentivised use; features that disincentivised use; and putative smoking cessation and/ or reduction. Putative cessation related to an ambivalence about how e-cigarettes were perceived as a potential mechanism for smoking cessation and/or reduction that proved largely unsuccessful in practice. S3 Appendix shows an overview of the analytic framework identifying themes and sub-themes that emerged during the analysis, with illustrative quotations additional to those presented below.

### Incentivising features of e-cigarettes

#### Indoor use and ease of concealment

Legislation banning indoor smoking in all workplaces has been in place in Ireland since 2004, and indoor smoking is considered strongly denormalised. E-cigarettes have not yet been regulated in the same way, and participants (n = 14) reported that being able to use e-cigarettes in places where smoking was not allowed was an incentive: *You could smoke anywhere you liked with them*, *well*, *before the restrictions came in* [John, M, HES]. Specifically, being able to smoke indoors was seen as a distinct advantage: *It was the novelty of being able to vape indoors and it was just easier* [FG, M, HES].

Indoor smoking was considered desirable and valuable, especially in the context of the climate and cold weather: … *you can smoke them indoors [because] that's a big factor*, *especially here in the cold* [Aine, F, HES].

The relative ease of concealment of e-cigarettes compared with pre-manufactured cigarettes (PMC) and roll-your-own cigarettes (RYO) was seen as a further advantage, making it more possible for young people to smoke indoors, as their parents or other adults would not smell e-cigarettes as they would PMC/RYOs. One student was allowed to smoke in his home but, because of greater parental supervision, his friend could not and so used e-cigarettes: … *my parents are usually*, *well they’re not usually in the house so I would find it easier to go and have a smoke*, *but his parents are always around so it’s just easier for him to have the vape* [Simon, M, HES].

#### Pleasant taste and flavour

Students (n = 11) mentioned the range of e-liquid flavours available and the taste of e-cigarettes (n = 8) as incentivising their e-cigarette use.

P4: *They taste a lot nicer than smokes*.
P5: *They tasted grand …*
P6: *The flavours can be real tasty* (others agree) [FG, M, HES].

#### Appearance

Participants (n = 11) noted the appearance of e-cigarette devices as a positive feature: *You can get them in different colours and all*, *it’s good* [Monica F, ESL], and also considered their similarity to PMCs (n = 7) as an incentivising feature of e-cigarette use.

#### Cost

Price (n = 8) and the long-lasting nature (n = 7) of e-cigarette devices were incentivising features of e-cigarette use for some young people in our study, both for college students *because it's much cheaper* [John, M, HES], and for early school-leavers: [I used e-cigarettes] *not to quit but to save money*. [Bradley, M, ESL]. Young people reported that e-cigarettes cost a lot less than PMCs or RYOs because the devices could be charged and re-used, thus saving money, and this motivated them to use e-cigarettes.

#### Other incentivising factors

Being able *to do tricks* with e-cigarettes: *It’s cool because you learn how to do tricks on them*. [FG, M, HES] was mentioned by 3 participants. Some other incentivising factors mentioned were immediacy of use, and not smelling like cigarettes: *You didn’t have to roll up*, *they didn’t make you smell*, *that was it really* [Simon, M, HES]. Feeling that it was ok to use e-cigarettes when children were present was also mentioned as an incentivising feature of e-cigarettes compared with PMC/RYOs: *There’s no smell off them or anything*. *There’s no smoke or anything so we were able to smoke them around the kids* [Davis, F, ESL].

### Disincentivising features of e-cigarettes

Overall, students noted many more negative features of e-cigarettes than positive ones, including negative user experience (n = 25) for themselves and for people close to them, inability to control the amount consumed (n = 19), and uncertainty and concern about health risks (n = 17).

#### Negative user experience

Negative user experience encompassed adverse health effects and unpleasant physical effects as well as unpleasant effects from problems with e-cigarettes devices themselves. Some participants who had tried e-cigarettes expressed a vague, generalised "dislike" of e-cigarettes

*Strange*, *like it's obviously not as strong … but I didn't really … like it* [John, M, HES].

*Unpleasant taste*. Although the range of flavours was considered an incentivising feature, participants also referred to unpleasant taste: *My ma tried to go on the e-cigarettes but I tried it and it was disgusting*. *I didn’t like it*, *yeah the taste was horrible* [Marnie, F, ESL], and among focus group participants:

P4: *It was just disgusting; I don’t like it*.
P1: *It tastes rotten*. [FG, F, ESL].

*Adverse health effects*. The strongest disincentivising feature of e-cigarette use mentioned by respondents was the adverse health effects that they report experiencing as a result of using e-cigarettes. These were primarily respiratory; students in their individual interviews reported that e-cigarettes affected their throats and made them cough:… *it just doesn’t agree with me throat or something like I dunno*… *like the minute I take a little drag I just start coughing me lungs up like it just*, *it’s horrible* [Caitriona, F, ESL]. Similar reports were made by female early school-leavers and higher-education students in their focus group interviews (S3 Appendix).

As well as their own negative respiratory experiences, participants also reported that they had observed friends and family experiencing negative respiratory tract reactions to e-cigarette use. Other adverse physical effects were reported in addition to lung and throat complaints, including becoming violently ill and headaches:

*P1*: *I got a migraine… I tried it twice and I tried two different types and I got the worst … and then about four months later I tried a different one … and the exact same thing happened* [FG, F, HES].

*Second-hand aerosol*. E-cigarette use by others was negatively described by some current smokers who were not current e-cigarette users. Female higher-education students in their focus group interview spoke about second-hand aerosol and the unpleasantness of having it blown in one’s face, indicating social stigma [50]:

P4: *And you’re just getting this like disgusting*, *sweet-smelling smoke blown at you*
P1: *It’s gross*.
P6: *I think that’s all the vapourisers are for though is just the whole smoke thing*, *it’s disgusting and pointless* [FG, F, HES]

And later:

P1: *It’s just this white smoke everywhere like*, *it’s obnoxious*, *I hate e-cigarettes* [FG, F, HES]
*Negative features of the device*. A further feature of negative e-cigarette user experience related to the devices themselves, a result of them being faulty or not working, and also being responsible for undesired outcomes: *My ma … got a fright when the liquid went into her mouth* [Marnie, F, ESL].

Respondents’ accounts of *things that go wrong with e-cigarette devices* were common, appearing to resemble a type of urban mythology:

P4: *… there has been stories …*
P4: *One blew up in a girl’s mouth before*.
P3: *And someone burnt a hole in his lung with it*. [FG, F, ESL]

Other problems with the devices were also mentioned, including losing parts of them, and the charger.

#### Inability to control amount

The capacity that e-cigarettes give users to continue using longer than they might wish or longer than they might feel comfortable doing, characterised by Hoek and colleagues as lack of satisfaction and failure of ending or completion [50], was a further disincentivising feature identified by 19 participants. Students suggested that they had little control over e-cigarette use in terms of amount, and certainly less control than they had compared with PMCs when they used them:… *there is no end to an electric cigarette*, *it’s a battery… you just keep going for ten minutes solid … So*, *I found that I would smoke more volumes of smoke with an e-cig …*[Simon, M, HES]. The male higher-education students returned to this topic more than once during their focus group interview:

P6: *But I feel like you smoke a lot more and it’s not as satisfying*.
P7: *Rollies it just finishes it and you can throw it away but with an e-cigarette you just keep on going*. [FG, M, HES]

Female early school-leavers both in individual interviews and focus groups also focused on the inability to control amount, leading to e-cigarettes being considered not strong enough, with respondents noting that it was difficult to get the right balance or strength in the e-cigarette user experience: … *like it felt like you didn’t even take a drag it was so light*. *And then she went onto a stronger one and that was way too strong so*, *you know* [Caitriona, F, ESL].

#### Uncertainty and concern about unknown and long-term health effects

The third major disincentivising feature of e-cigarette use related to potentially adverse health effects (n = 25), particularly the potential long-term, but as yet unknown, harmful effects (n = 17), and concerns about addiction (n = 8). Respondents noted that potential health effects might be currently unknown but expressed uncertainty and concern regarding the content of e-cigarettes as well as about their effects. They pointed to a lack of *legitimate* scientific studies and also to the role of the tobacco industry in how messaging about e-cigarettes and tobacco product is carried out.

Students had many concerns about e-cigarettes, including in comparison with other tobacco products. These included concerns about the relative newness of e-cigarettes compared with other tobacco products:

P1: *I think because like they only came out in the last few years…* [FG, M, HES]

about how harmful they might be, especially in the long-term:

*I think there are a lot of unknowns with the e-cigarettes as well*, *like how harmful they can be LT [long-term]*. [Aine, F, HES]

and about the ingredients in e-cigarettes:

*I looked into the juice*, *the glycerol*, *and all that kind of stuff and I think there are ingredients that go into anti-freeze that go into e-cigarette juice*. [Simon, M, HES].

Students drew attention both in individual and in focus group interviews to the unknowability of the long-term effects because the products were relatively new and because there was an absence of research on the long-term effects of e-cigarette use:

P1: *… but there are very few legitimate scientific studies on them on the long-term effects of them*. [FG, M, HES].

### Putative smoking cessation

The third theme to emerge from categoric data analysis was named putative smoking cessation because although use of e-cigarettes to aid smoking cessation or reduction was mentioned by participants (n = 15) as a motivation for use, only one participant said that she had successfully used e-cigarettes to give up smoking

P6: *That’s how I quit smoking* [FG, F, ESL RYO].

Even this person was possibly unsuccessful in giving up smoking cigarettes, as the interviewer noted that:

P6 *had tried to give up…well*, *with smokes in her pocket*.

Only one respondent (out of 62 in total) reported using e-cigarettes to reduce cigarette smoking:

…*and at the time*, *I switched from Amber Leaf back to John Player and I went through nearly a pack a day and when I went onto the e-cigarettes*, *I went from a pack to a pack every five days*, *so I cut down a good bit yeah*. [Bradley, M, ESL]

Therefore, while students described using e-cigarettes for smoking cessation and/or reduction, they also described their almost complete lack of success in doing so.

Questioning about smoking cessation formed part of the interview schedule and so was discussed in all individual and focus group interviews. Young people appeared keen to stop smoking and, in the focus group interview with female, Youthreach students, they identified factors other than e-cigarettes that might help them to quit, being particularly aware of the importance of peer group in continuing to smoke:

I: *And what do you think you would need to do to stop smoking*?
P4: *Pick up a hobby*.
P5: *Having more things to do like*.
P6: *Not being around people who are smoking*. [FG, F, ESLRYO]

Respondents' descriptions of e-cigarettes suggested that they believed that e-cigarettes were a known or recommended means of smoking cessation or reduction (of PMCs or RYOs):

..*like they’re meant to get you off them* [Monica, F, ESL].

A rationale for using e-cigarettes to quit smoking was their perceived *relative healthiness* compared with PMCs and RYOs. For example, for a time, Monica used e-cigarettes to try to quit smoking. While all her friends used RYOs or PMCs, and while she was ambivalent about the authenticity of e-cigarettes, she was convinced of their relative health benefits:

*It just felt weird*, *me there with a fake smoke in my hand*, *and they’re there with a smoke but when I thought to myself*, *my lungs are going to be ok*, *and your lungs are going to be rotted*, *do you know what I mean*? [Monica, F, ESL].

Students identified e-cigarettes as one of a number of smoking cessation approaches that might be tried. They reported, however, that e-cigarettes were not any more successful than other smoking cessation methods:

*You see*, *the way I started on vapes was I wanted to give up smoking and I tried the gums*, *I tried the sprays*, *I tried the patches and I tried cold turkey and they weren’t working like*. *You don’t get the same satisfaction*. *So*, *I said*, *I’ll try the vapes*, *and they don’t work either so I went back onto the rollies again*. *I haven’t smoked it (e-cigarette) for about a month*. [Simon, M, HES]. Later, when probed further (Interviewer: *And why don’t you not find enjoyment out of it anymore*?), Simon expressed anxiety about the strong negative impact of his smoking on his lungs, making his inability to quit more worrying: *It doesn’t work anymore*, *and I can feel it having an impact*. *My lungs feel like concrete when I wake up some mornings* [Simon, M, HES].

Dual use of e-cigarettes and either PMCs or RYOs was mentioned by participants in individual and focus group interviews, speaking about themselves and about friends or family members:

P4: *My ma has a pink one*, *she loves it*, *but she smokes as well* [FG, F, ESL]. Another participant in a focus group [F, ESL) described her mother simultaneously using PMCs and e-cigarettes, i.e. having a cigarette in one hand and an e-cigarette in the other:
P6: …*I’m not even joking like*, *that electric smoke is not out of her hand*. *She’d light a smoke and th*en…
I: *So*, *she would be using a cigarette and…*
P6: *Yeah like her smoke would be in her hand and then the other one is in the other hand*. *She’d be smoking the two of them at the same time*. *So*, *she’s actually using it*, *or like doubling it because she’s using an electric smoke*, *and she’s using the smoke*. *Like it’s not stopping her from smoking*, *because she’s smoking the same amount [of cigarettes] that she used to* [FG, F, ESL].

Because e-cigarettes could be used indoors they led to more extensive use (*constantly puffing*), and were seen as antithetical to quitting, especially when dual use occurred. Áine [F, HES] described her friend who used e-cigarettes as a smoking cessation aid:… *a close friend of mine tried to wean her way off tobacco with them but I found she was constantly on it more so than if she was smoking tobacco*, *you know like*, *they’re constantly puffing it because you can constantly do it indoors as well*. *I think you’re more inclined to be doing it*. Thus, dual use was part of the process of unsuccessful quitting. Áine’s friend was ultimately unsuccessful in using e-cigarettes to quit: *She’s off the e-cigarettes and has just gone back to the rollies* [Áine, F, HES].

Participants summarised many of the reasons for not using e-cigarettes as a smoking cessation aid, including dual usage, cross addiction, potential damage caused by e-cigarettes, lack of research on e-cigarettes, the *synthetic-ness* (inauthenticity) of e-cigarettes (compared with tobacco) (See S3 Appendix for more complete quotes).

## Discussion

Although smoking prevalence among young people in Ireland has been declining since a high of 41% in 1995, e-cigarette use has continued to rise. Relatively few qualitative studies—and none in Ireland–have sought to understand why young people use e-cigarettes. This study adds to the literature by using qualitative methodology to explore young people’s views about, and experiences of, e-cigarettes in Ireland. In-depth individual and focus group interviews with 62 young smokers aged 18–22 years provide a picture of young people’s real-world use of e-cigarettes and their views about e-cigarettes. The themes identified in our data analysis can help us to understand what features of e-cigarettes young people find desirable and motivate them to use them, and also what features disincentivize young people’s use. The first two of the three major themes reported are incentivising and disincentivising features of e-cigarette use. Incentivising features included price, appearance, taste/ flavours, and the possibility of indoor use, confirming findings from other jurisdictions [14–18, 23, 24, 31]. The absence of the “tell-tale odour” [16] of combustible cigarettes was also noted by participants. Like others [14, 15, 18, 24, 31], we find that price is a key driver in use of e-cigarettes and should be factored into tobacco control policies.

Disincentivising features related to adverse health effects (pain, discomfort, sore throat, coughing, headache) and unpleasant physical effects (bad taste and problems resulting from device faults). Over-consumption of e-cigarettes arising from inability to control intake (too easy to smoke too much compared with PMCs and RYOs) and the indefinite ending or completion of usage [50] was also a disincentive, confirming similar findings by others [15, 50]. Despite some evidence of “ostentatious differentiation” [50] as regards e-cigarettes vis-à-vis “real” cigarettes, like Lucherini et al. [14], we noted that similar performativities [51] regarding smoking and e-cigarette use led to participants’ disapproval about elements of smoking practices (such as clouds of smoke/aerosol in public places) being extended to e-cigarette practices.

Concerns about "greater addictiveness", product taste, and negative features related to the product itself, such as device faults were also disincentives. Repeated nicotine use and its role in addiction is of broader interest in tobacco control research [15] and young people’s use of e-cigarettes merits further research in this regard. We agree with McDonald and Ling’s (2015) insight that participants used their own bodily sensations as a source of information about the relative safety or risk of e-cigarettes but while they found that participants did not have major health concerns about these products, feeling comfortable to experiment with e-cigarettes [15], we found that young people had great concerns about potential adverse health effects, particularly those that were as yet unknown and that could occur with longer-term usage. We found somewhat more certainty about the negative health consequences, lack of safety, and regulatory oversight of e-cigarettes compared with the relative uncertainty about these reported by others [23, 24] who found there was confusion about whether e-cigarettes are “healthier” than smoking. While others [14, 18] have found that absence of information about the relative harms of e-cigarettes may lead young people to form positive opinions about e-cigarette use, young people in our study were less convinced. Rather, overall, they were more negative than positive about e-cigarettes.

The third major theme to emerge from the data concerned what we called putative smoking cessation and reduction. Putative cessation refers to the conflict in participants' views between their expected use of e-cigarettes as a smoking cessation or reduction aid and their observed reality of e-cigarettes’ failure in this regard, with reported outcomes including: failure to quit or reduce leading to continued or resumed PMC and/or RYO smoking; dual use of e-cigarettes and other tobacco products; and inability to quit e-cigarettes.

Researchers in England [24] found that e-cigarettes were used as therapeutic aid to stop or cut down smoking among adult users (median age 44 years), but we did not find this to be the case with young people in our study. Although smoking cessation/ reduction was mentioned as a reason for starting to use e-cigarettes, we considered this motivation to be putative because young people in this study were almost entirely unsuccessful in using e-cigarettes to quit or reduce smoking. Thus, unlike others [17], we found that e-cigarettes were not successfully used to replace combustible cigarettes. More likely routes from e-cigarette use were quitting e-cigarettes in favour of smoked tobacco, and continuing using of e-cigarettes as well as smoked tobacco (dual use).

Many quantitative studies have found that e-cigarette use depresses smoking cessation [52]. As regards adolescents, earlier quantitative research found that e-cigarette use does not discourage, and may encourage, conventional cigarette use among US adolescents [53]. Furthermore, those who had made an attempt to quit smoking were more likely to use e-cigarettes but less likely to no longer use cigarettes [54], perhaps because some adolescents respond to advertising and marketing claims that position e-cigarettes as a smoking cessation aid. Our qualitative study confirms these findings.

The creation of associations between e-cigarettes and “friendly and ubiquitous personal electronic devices rather than deadly and stigmatised tobacco products” together with social media campaigns may facilitate e-cigarette use by young people [16]. Young people in our study recognized such efforts to attract them to e-cigarette use through advertising imagery and sponsorship at the local level. Given the focus of e-cigarette marketing strategies on young people and on ensuring that e-cigarette products appeal specifically to them through emphasis on lifestyle [11], participants in this study demonstrated a surprisingly critical consciousness about the use of celebrities, exotic locations and competitions to appeal to them. Further, they were even somewhat cynical in their descriptions, including calling out the possibility of tobacco industry involvement in encouraging action with such potentially negative consequences. This finding suggests that the inclusion in tobacco-related health education of critical literacy in reading advertising, marketing, and social media campaigns could offer benefits.

Hoek et al. have already established in New Zealand that strategies used by the e-cigarette industry such as event sponsorship, alliances with youth-oriented brands and role model endorsers, are comparable to those used by the tobacco industry, suggesting the opportunity to counteract e-cigarette marketing strategies, as has been done in countering the tobacco industry strategies [11]

### Implications for stigmatisation/denormalisation

The strategy of ‘denormalising’ tobacco use has been one of the cornerstones of the global tobacco control movement for at least 20 years [55]. We know that one effect of denormalization is that it makes visible smoking more difficult for people because of the perceived likelihood of public disapproval for an action that is not considered socially acceptable. Unlike previous studies that found mostly positive reactions to e-cigarettes [18], young people in our study were keenly aware of the social unacceptability of RYO use resulting from denormalisation of tobacco use in Ireland [56]. Equally, conversely, they saw e-cigarette use as a means of combatting the denormalisation of smoking, because a key incentivizing feature of e-cigarette use was being able to smoke them anywhere, including in places where smoking tobacco was not allowed, and especially being able to use e-cigarettes indoors. We confirm the work of McDonald and Ling and others that an attractive feature of e-cigarettes is their use in smokefree spaces, and that e-cigarettes may also “renormalise (what appears to be) the act of smoking in smoke-free spaces” [15]. However, we observed also, as others have [50] that e-cigarette use was not without stigma, with participants reacting adversely to cloud plumes and commenting on the unpleasant smell of second-hand aerosol. We note, following Bell and colleagues, the potential use of stigma as a public health tool, which may be particularly powerful when designing interventions for young people [55].

Hoek and colleagues found that young adults used e-cigarettes to construct rituals that recreated or replaced smoking attributes [50]. We did not find particular evidence of use of e-cigarettes for construction of rituals, although our earlier work with this sample of young adults suggested that ritual played a significant role in their use of RYO products, suggesting differing motivations among these young people for choosing different tobacco products [56].

E-cigarette use may represent a significant threat to tobacco control through the undermining of denormalisation, even or especially in societies which are strongly denormalised. This may occur, in part at least, through a paring back of what Chapman and Freeman (2008) call the ‘spoiled identity’ [57] of smokers, operationalized in many markers visible among Australian tobacco users. Smokers’ spoiled identities have been compounded by the ‘relentless tide of bad news’ [57] about smoking carrying numerous subtexts about smokers as undereducated, malodorous, excessive users of public health services, selfish and thoughtless, and as employer liabilities. Like Australia, Ireland is a country with progressive tobacco control legislation and regulation, and smoking is banned in all workplaces and increasingly in outdoor areas where there are children as in playgrounds, as well as on public transport. Participants in our study spontaneously referred to several markers of the spoiled identity of smokers, including smokers as malodorous, as unattractive and undesirable housemates, and as addicts. Compared with tobacco use, e-cigarette use was seen as an antidote to these markers, including e-cigarettes users being less malodorous than users of smoked tobacco, and being able to use e-cigarettes around other household members, including children, without fear of reproval. Thus, e-cigarettes offer the possibility of reducing stigmatization associated tobacco use in jurisdictions with strong tobacco control regulation and denormalization. This topic merits further research, particularly qualitative research that could ‘drill down’ into users’ beliefs and perceptions about e-cigarette use, stigmatization, and denormalization.

### Implications for policy and legislation/regulation

Ireland, as an European Union (EU) Member State, is subject to EU Directives. One such Directive, the Tobacco Products Directive 2014/40/EU [58] in its Article 20 focuses on regulation of e-cigarettes and had not been transposed into Irish law when this study was carried out. Also the choice and range of devices was limited, consisting mainly of cigalikes and pens. Article 20 enforces implementation of many rules such as maximum nicotine concentration and volume for cartridges, tanks and nicotine liquid containers and safety features such as child-proof containers. However, the TPD does not deal with access. At present children may buy devices although some outlets have a voluntary restriction. The Irish Government has proposed further legislation to regulate e-cigarette use which is planned to come before the next Parliament. This study offers several considerations for e-cigarette regulation. First, access to e-cigarettes was reported by participants in our study to cause no difficulty, being readily available from corner shops, supermarkets, family members and friends; this finding is not unusual and suggests that regulators should limit the availability of e-cigarettes to licensed outlets. It is also proposed that 21 years of age be the legal age for access. The use of e-cigarettes where smoking is forbidden was offered as a clear incentive for their usage which we recommend be changed so that the same laws governing Smokefree should apply to e-cigarettes. Price was also a significant incentivising feature that demands action while making the use of e-cigarettes available at affordable prices for adult smoking cessation. The failure in this group to find e-cigarettes helpful for cessation demands further study to establish whether this is generalisable and to pay attention to this aspect of all cessation trials where e-cigarettes are used, by careful monitoring of such trials involving this age group.

Among adult users, flavours play a major role in e-cigarette initiation for current smokers, former smokers and vaping-susceptible non-smokers, and remain important to those who continue using e-cigarettes [59]. In our study, flavouring in e-cigarettes was mainly a positive feature, contributing to favourable perceptions and motivating use for some participants. A few participants, however, were derogatory about many e-cigarettes flavourings, disliking the taste of particular flavours and rejecting e-cigarettes because e-cigarettes flavours did not taste like ‘real’ cigarettes, to which they were addicted, a further example of “nostalgic dissatisfaction” [50]. It is clear that flavouring is being used as a marketing tool that needs further regulation to prevent e-cigarette uptake in young people.

Perhaps the most salient feature of e-cigarette use in terms of regulation is their constituent elements. Little or no information was available in Ireland regarding the ingredients/ constituents in/of e-cigarettes when this study was carried out, and this was a topic about which young people in our study expressed concern and cynicism. Article 20 of the TPD should alleviate this problem. They were sceptical about e-cigarettes being “supposedly more healthy” than pre-manufactured cigarettes given that very little was known about the long-term effects of the different e-cigarette components. This suggests that a two-pronged approach in terms of constituents of e-cigarettes may be helpful in reducing e-cigarette usage among young people. Since, in the EU, manufacturers are now obliged by regulation to report the constituents of their e-cigarettes products in full and, as young people are suspicious of and negative about, the array of constituent products in e-cigarettes, this information and its use in health education programmes aimed at reducing e-cigarette usage among young people offers a prospect of success.

As others have noted, there may be a lack of information from government agencies on the health risks of e-cigarettes [60], and, particularly for young people, we recommend that clear, evidence-based scientific information be made available in guidelines and incorporated into tobacco-related health education for primary, secondary and tertiary education students.

### Smoking cessation

Young people in this study were aware of the reputation of e-cigarettes to assist in smoking cessation and/or reduction and made reference to that expectation. They were somewhat sceptical, however, about the veracity of claims made about e-cigarettes as a smoking cessation aid, using qualifiers such as “they are supposed to help” and seeming to doubt. Most telling were their reports of their own failure to quit, and the failure of people they knew to quit. Even in the process of trying to quit, their experiences were of dual use and adverse physical effects from e-cigarettes. They reported wanting to quit smoking and so they had tried e-cigarettes. Indeed, microsimulation experiments suggest that the social influence of the prevalence of e-cigarette use may influence young people to initiate or continue conventional cigarette smoking [61]. The desire of young people to quit tobacco should be addressed and services tailored to their needs as their uptake of presently available services is low [62].

Dual use among adults is not homogenously practiced. For example, Robertson and colleagues found that practices developed in multiple ways, including as a means of managing perceived “inauthenticity” of e-cigarette use compared with smoking; through rationalising decreased tobacco use rather than smoking cessation as “success”; as a way to reduce the financial burden of smoking and to circumvent smoke-free policies; and as an attempt to comply with social group norms and manage stigma [31]. When we explored e-cigarettes and smoking cessation, we found evidence of dual use but did not gain insight into its complexity, neither routes into it, nor continuing practices and we recommend further qualitative research to elucidate this.

### Methodological limitations

We acknowledge limitations to our study, especially in terms of the study’s qualitative methodology and its relatively small and somewhat homogeneous sample of 62 young people in one city, Dublin, Ireland. At the same time, given the marked increase in e-cigarette usage in this age group, a qualitative approach is relatively under-used in research on e-cigarette use in Ireland. Young people’s perspectives on, and experience of, e-cigarette use are also under-researched, such that this qualitative methodology allows for new insights, understandings, and meanings [63] about e-cigarette use, at a time when it is growing, largely unregulated, across the world.

### Contribution

There is a gap in qualitative research that would provide more nuanced and detailed accounts of young people's views on, use of and experiences of e-cigarettes, particularly in how they report e-cigarette use in relation to smoking cessation. We feel that identifying disincentivising factors associated with e-cigarette use may be helpful in countering some of the inducements for initiation in this group. Also, a comprehensive understanding of how these products incentivise and disincentivise use is important so that strategies can be developed to discourage their use by young people. The clear finding from this qualitative study is that e-cigarettes are neither successfully used for smoking cessation nor, ultimately, seen as useful in that regard by young people.

## Conclusion

Students in our study reported far more negative features of e-cigarettes than positive ones. We recommend incorporating this in policy interventions aimed at reducing use in young people. In addition to reporting adverse health effects and negative physical responses, a degree of scepticism about the "health benefits" of e-cigarettes was evident. Among young people in our study, e-cigarettes were neither successfully used for smoking cessation nor seen as useful in that regard. Legislation, regulation, and denormalisation have clear roles to play in whether and where young people use e-cigarettes.

## Supporting information

S1 Appendix Timeline of tobacco control policies and interventions in Ireland 1995–2020. (DOCX)

S2 Appendix Individual and focus group interview schedule. (DOCX)

S3 Appendix Thematic analytic framework. Themes and sub-themes of e-cigarette users’ accounts of e-cigarette use, with illustrative quotes.
(DOCX)

S1 Table Demographic, smoking and e-cigarette use: Characteristics of study participants. (DOCX)
