# Supplementary material for: A qualitative study of e-cigarette use among young people in Ireland: Incentives, disincentives, and putative cessation
Source: PLoS One. 2020 Dec 28;15(12):e0244203. doi: 10.1371/journal.pone.0244203 (PMC7769428; doi:10.1371/journal.pone.0244203)
Supplement: S1 Appendix — (DOCX) [file pone.0244203.s001.docx]

**S1 Appendix: Timestamps of Tobacco Control Policies in Ireland, 2000-2015**
