# Supplementary material for: A qualitative study of e-cigarette use among young people in Ireland: Incentives, disincentives, and putative cessation
Source: PLoS One. 2020 Dec 28;15(12):e0244203. doi: 10.1371/journal.pone.0244203 (PMC7769428; doi:10.1371/journal.pone.0244203)
Supplement: S3 Appendix — Themes and sub-themes of e-cigarette users’ accounts of e-cigarette use, with illustrative quotes. (DOCX) [file pone.0244203.s003.docx]

# S3 Appendix

# Title. Thematic Analytic Framework. Themes and sub-themes from participants’ accounts of e-cigarette use, with illustrative quotes.

Legend for Student Comments:

*Individual interviews*: Participant pseudonym; M/F (male/female); ESL (Youthreach= Early School Leaver) or HES (Higher Education Student)

*Focus Group interviews*: P1 – P8 (Participant 1, Participant 2, etc.); M/F (male/female); ESL (Youthreach students = Early School Leavers) or HES (Higher Education Students)

Ps = other participants, speaking together, often expressing agreement.

| **Theme** | Student comments (Extracts from individual interviews and focus group interviews) |
| --- | --- |
| 1. **Incentivising Features** |  |
| *Indoor Use / Ease of concealment* | *You could smoke anywhere you liked with them, well before the restrictions came in*. [John, M, HES]  *It makes smoking inside easier I suppose.* [Simon, M, HES]  *It was the novelty of being able to vape indoors and it was just easier*. [FG, M, HES]  *Well, I'd say that it has a lot to do with the fact that you can smoke them indoors because that's a big factor, especially here in the cold*. [Aine, F, HES]  … *my parents are usually, well they’re not usually in the house so I would find it easier to go and have a smoke, but his parents are always around so it’s just easier for him to have the vape*. [Simon, M, HES] |
| *Taste and Flavour* | *I* *got blueberry, cherry, bubble-gum, good. The fact that I liked about them is that you can get different flavours than the e-cigarette. That way I could actually taste something, instead of tasting smoke, that’s what really encouraged me to stay on it*. [Monica F, ESL]  P4: *They taste a lot nicer than smokes.*  P5: *They tasted grand …*  P6: *The flavours can be real tasty* (others agree).  P5: *Yeah vanilla, coffee, chocolate and shit.*  P4*: Yeah vanilla.*  P6: *Yeah blueberry, all the fruity ones.*  P4: *My mate in his room has got literally got a stand which is just covered in bottles and there is easy like 15 or 20 bottles of different flavours*. [FG, M, HES]  P4: *Yeah, if someone had an e-cig and they have like flavours and stuff, yeah, you’d try it, you know Eddie’s… vanilla… mango…* (laughter). [FG, M, HES]  P5: *O… watermelon!* [FG, F, HES]  P6: *The flavours in them are alright, they taste nice.*  P3*: … tar and all.* [FG, M, ESL].  *The raspberry one is nice; I don’t like the toffee one or anything. There are all these different ones if you know what I mean?* [Pascal, M, ESL]  *I thought the taste of them was actually nice. Like some of them have different tastes and different flavours off them.* [Tom, M, ESL] |
| *Appearance* | *You can get them in different colours and all, it’s good*. [Monica F, ESL]  P2: *Like my brother has a nice one, its steel and its glass and its see-through for the things that you put your lips on. It looks good it does.* [FG, F, ESL]  P2: *It’s becoming a more of a fashion statement now more than anything*  Interviewer: *How do you mean fashion statement?*  P2: *It’s kind of a thing that they look like that they want to be smokers but they don’t want the whole...* (voice trails off). [FG, M, HES] |
| *Cost* | *… because it's much cheaper.* [John, M, HES]  [I used e-cigarettes] *not to quit but to save money*. [Bradley, M, ESL]  *… the fact that you can charge it... That’s about it... It’s a money saver, saving your money.* [Albert, M, ESL] |
| *Other incentivising features* | *It’s cool because you learn how to do tricks on them.* [FG, M, HES]  *For me, it’s almost turned into a hobby. I’d nearly see myself as a hobbyist for an e-cig now rather than as a casual smoker or a vaper. I don’t bring it out but I enjoy, I don’t want to go to in-depth but there are loads of things that you can do with the coils so determining how you position the coil or how much coil you use determines how much flavour or how much cloud you get. It’s all confusing stuff but it’s a lot of fun to look up.* [Ryan, M, HES]  *You didn’t have to roll up, they didn’t make you smell, that was it really.* [Simon, M, HES]  *There’s no smell off them or anything. There’s no smoke or anything so we were able to smoke them around the kids.* [Davis, F, ESL]  *It just felt weird, me there with a fake smoke in my hand, and they’re there with a smoke but when I thought to myself, ‘my lungs are going to be ok, and your lungs are going to be rotted, do you know what I mean?’ That’s the way I thought about it.* [Monica, F, ESL] |
| 1. **Disincentivising Features** |  |
| *General negativity*  *Unpleasant physical response: Taste* | *Strange, like it's obviously not as strong, as you're just smoking vapour but I didn't really get the hang of it or I didn't really like it.* [John, M, HES]  Interviewer: *So generally how would you describe your experience of using the vape? …*  P: *Negative, it’s still smoking like*. [Pascal, M, ESL]  *It didn’t taste nice at all, like at all.* [Damien, M, ESL]  *My ma tried to go on the e-cigarettes but I tried it and it was disgusting. I didn’t like it, yeah the taste was horrible.* [Marnie, F, ESL]  P4: *It was just disgusting; I don’t like it.* [FG, M, HES]  P1: *It tastes rotten.* [FG, F, ESL] |
| *Unpleasant physical response: Adverse respiratory and other health effects* | *Yeah well it affects me … breathing…  Eh, I don’t like them… Just the feeling it has on me Adam’s apple at me throat like* [Albert, M, ESL]  *... it just doesn’t agree with me throat or something like I dunno ... like the minute I take a little drag I just start coughing me lungs up like it just, it’s horrible.* [Caitriona, F, ESL]  *It was a bit strong. Like you would have to get used to it, for the first few days we were coughing and stuff. It’s very harsh on your throat.* [Davis, F, ESL]  *No, I didn't like it; I just coughed a lung up!* [Quinn, F, HES]  P4: *No, I think it’s like sand in your throat or something, it’s a real horrible feeling.* [FG, F, ESL]  P5: *Yeah, it really hits the back of your throat, e-cigarettes, I think.*[FG, F, HES]  P3: *It just hits your throat and it’s horrible.* [FG, F, ESL]  *One of my friends had a shot of it, and thought it was deadly. She said that she might go on it, but she didn’t. She thought it was very harsh on the throat and didn’t like it at all.*[Davis, F, ESL]  P1: *I got a migraine, I tried it twice and I tried two different types and I got the worst-! I don’t suffer from migraines and I had this smoke, well e-cigarette thing, and I had to go- about half an hour after I had to go to bed and all the lights had to be off like, I was suffering really really badly from it, and I was confused, I was like what was that?! And I was like ‘Oh it might have just been something else’ and then about four months later I tried a different one ‘cos it was a different flavour, ‘cos the first one I tried was blueberry and the other one was like I think it was watermelon, I hate watermelon, and I tried it anyway and the exact same thing happened. And I was like right well they obviously don’t agree with me*. [FG, F, HES]  P2: *One of my dad’s friends was violently ill for about a week off one of those things.* [FG, M, HES]  P4: *I took a drag off one and it nearly flung me back to the wall; I was like ‘get that away from me’.* [FG, F, ESL]  *… and now they are running into health problems because it affects the throat. It’s a hot liquid going down your throat as steam.*  [Daisy, F, HES] |
| *Unpleasant physical response: Secondhand aerosol* | P1: *Billowing out these clouds of smoke in your face like…* [FG, F, HES]  P4: *And you’re just getting this like disgusting, sweet-smelling smoke blown at you.* [FG, F, ESL]  P1: *It’s gross.*[FG, F, HES]  P6: *I think that’s all the vapourisers are for though is just the whole smoke thing, it’s disgusting and pointless.*  P1: *It’s just this white smoke everywhere like, it’s obnoxious, I hate e-cigarettes.* [FG, F, HES] |
| *Inability to control amount* | *… there is no end to an electric cigarette, it’s a battery, once it’s gone, it’s gone, otherwise there is no end to it, you just keep going for ten minutes solid, whereas with a smoke, once it’s gone, it’s gone. So, I found that I would smoke more volumes of smoke with an e-cig, so it wasn’t really helping me that way.* [Simon, M, HES]  *When I was smoking that now I did find that I was smoking it heavily. And constantly. ‘Cos it just wouldn’t go down and d’ya know you couldn’t see it evaporating or anything so yeah*. [Albert, M, ESL]  P6: *But I feel like you smoke a lot more and it’s not as satisfying.*  P7: *Rollies, it just finishes it and you can throw it away but with an e-cigarette you just keep on going.*  P3: *You just don’t stop and you go through a whole thing of liquid.* [FG, M, HES]  P4: *They made my chest feel really heavy. I don’t get that with smokes but after vaping you kind of feel like when you get a chesty cough and you feel that there is pressure down on your chest, I feel like that if I vape for a while but I don’t feel like that after I smoke.*  P2: *Its cos you are vaping for so much longer though.*  P6: *Yeah cos you just keep pulling on it.*  P1: *There is no end.*  P5: *Yeah with a smoke there is a set amount. Like you roll it, you smoke it and you’re done.* [FG, M, HES]  P6: *With the e-smokes, you don’t know when to stop, like you don’t know when to stop smoking. Like a smoke goes down and you tap it out, but with that, there’s no tapping out of it.* [FG, F, ESL]  P4: *Yeah like you just press a button, it doesn’t even seem like a smoke.*  P3: *You can just smoke it as much as you want.*  P6: *You don’t know when to stop, I think that’s the problem with them.* [FG, F, ESL]  *...like it felt like you didn’t even take a drag it was so light. And then she went onto a stronger one and that was way too strong so, you know.* [Caitriona, F, ESL]  I: *Yeah yeah. Ahm, so when you were smoking it did you find you were smoking cigarettes less at all?*  A: *When I was smoking that now I did find that I was smoking it heavily. And constantly. Cause it just wouldn’t go down and d’ya know you couldn’t see its evaporating or anything so yeah.*[Albert, M, ESL]  P4: *They made my chest feel really heavy. I don’t get that with smokes but after vaping you kind of feel like when you get a chesty cough and you feel that there is pressure down on your chest, I feel like that if I vape for a while but I don’t feel like that after I smoke*  P2: *Its cos you are vaping for so much longer though*  P6: *Yeah cos you just keep pulling on it*  P1: *There is no end*  P5: *Yeah with a smoke there is a set amount. Like you roll it, you smoke it and you’re done.* [FG, M, HES]  Interviewer:  *Em, so how often were you smoking it when you had it?*  R: *Eh, probably more than when I was smoking the cigarettes like.*  Interviewer:  *Ok, why was that?*  R:  *I dunno, I suppose I wasn’t getting the satisfaction off it so I was smoking it more …* [Ron, M, ESL] |
| *Lack of information about products/ Concern about unknown and long-term health effects* | P1: *I* *think because like they only came out in the last few years…* [FG, M, HES]  *I think there are a lot of unknowns with the e-cigarettes as well, like how harmful they can be LT* [long-term]*.* [Áine, F, HES]  *I looked into the juice, the glycerol, and all that kind of stuff and I think there are ingredients that go into anti-freeze that go into e-cigarette juice.* [Simon, M, HES]  *... I’m still not 100% sure about (the health benefits) because there are a lot of substances that they use in those that haven’t been fully tested* ... [Finn, M, HES]  P1: *Yeah, they were saying like they’re healthier, they’re making it out like it’s healthier for you.*  P2: *Nobody actually knows if it’s healthier for you … The vapour is supposed to be worse for you, you don’t know what’s in them like, you know what I mean? Anything could be in them things.* [FG, M, ESL]  P1: *I think because like they only came out in the last few years but there are very few legitimate scientific studies on them, on the long-term effects of them.*  P4: *Yeah, because they haven’t been around long-term…*  P1: *Once science comes out and says either they’re actually as bad and yes, you are still as addicted to nicotine, and people have this idea that it’s better than smoking, it might be, but you don’t know.* [FG, M, HES]  P6: *Plus, they did a study recently that shows that e-cigarettes are a lot worse for you because the smoke goes down a lot quicker, so it’s like…*  P4: *It’s like popcorn lung or something?*  Ps: *Yeah.*  P6: *So, it’s a lot easier to get a lot of severe side effects faster than if you smoked regular cigarettes, so it’s like, so much worse for you, and people think like Oh, it’s so healthy I’m going to quit on this like.*  P3: *‘Cos it’s like they didn’t do any long- term studies on it or anything like that.* [FG, F, HES]  P5: *Yeah, and even when you do hear that they’re bad, you can’t be sure that it’s not tobacco companies lobbying to get…* [FG, M, HES]  P1: *Yeah, like at the end of the day, they’re all bad. Like you’re dosing yourself in addictive substances.*  P4: *Yeah, like you shouldn’t be doing that to your lungs*. [FG, M, HES]  P1: *I feel like again… there hasn’t been long-term studies done on this, like whatever about smoking, everyone knows it’s bad for you, people have been smoking for hundreds of years but they haven’t been inhaling this gel for like 40 years so...*  P5: *Yeah you don’t know what it is.*  P1: *Yeah you don’t know, in 20 years’ time when- like the lads that smoke in* [name of college]*, they’re only kids like, they’re fresh out of secondary school and they’re smoking these vapes, you wouldn’t know, your lungs at 30 could be absolutely destroyed like. No one knows if that’s going to happen yet, so for me alone like, I’d be like look I want to give up one bad habit, I’m not going to take up something that could like possibly really badly affect my health in the future.*  Ps: *Yeah, yeah.*[FG, F, HES]  P2: *It’s too synthesised as well. When you have tobacco, you’ve got this like, as you said, fresh stuff…*  Ps: *Yeah, yeah.*  P1: *Yeah, it feels more natural.*  P5: *Yeah, it has something natural in it at least, like something that comes back from the tobacco plant itself.*  P2: *The earth.*  P5: *Instead of it’s like synthesised gel that’s like completely plastic, ‘cos you are essentially smoking plastic once you break it down.*  P4: *A polymer.*  P5: *Sorry!* (laughter)  P1: *Yeah knowing science really helps!* (laughter) [FG, F, HES]  *I think, if you’re coming from conventional cigarettes which have, I actually done a project on lung disease and the vast majority of lung disease, like 90% is caused by smoking, and I think something like over 200 toxins in normal cigarettes so it’s like six of one, half a dozen of the other but it’s still very concerning that you don’t actually know what’s in there, you just know that there is nicotine and flavourings but there could be countless other chemicals.* [Paul, M, HES] |
| *Negative features related to the device* | *My ma wanted to come off the smokes because it would be cheaper but she got a fright when the liquid went into her mouth, so she got off that and back onto the smokes.* [Marnie, F, ESL]  P4: *Supposedly its actually healthier than smoking but I just wouldn’t trust it because there has been stories...*  Interviewer: *And why wouldn’t you trust them?*  P3: *Because of all the stories that are coming out about them.*  P4: *One blew up in a girl’s mouth before.*  P3: *And someone burnt a hole in his lung with it.*  Interviewer: *So, you’re scared of it blowing up?*  P4: *Yeah, it’s all that electronic stuff going down your lungs then.*  [FG, F, ESL]  P4: *I remember I took a drag of my ma’s one and the oil poured all over me like, the oil was dripping.* [FG, F, ESL]  P3: *That’s what put me off them like.*  Interviewer: *So, it was leaking?*  P4: *Yeah but it was in my mouth like.* [FG, F, ESL]  *The reason I quit the e-cigarettes is because I kept losing the white bit. Because you have the white bit and the orange bit. The white bit is like the battery. I kept losing that and I kept having to buy a new one and I just stopped it altogether.* [Bradley, M, ESL]  P2: *I have one [e-cigarette] but I lost the charger off it*. [FG, F, ESL] |
| 1. **Putative Cessation and Reduction, including relapse and dual use** |  |
|  | Interviewer: *Why did you get it* [e-cigarette]?  *A: Just to try stop smoking, like to try stop smoking altogether.*  Interviewer: *To try quit?*  *A: Yeah yeah. But it just didn’t happen*. [Albert, M, ESL]  P6: *That’s how I quit smoking.* [FG, F, ESL]  Interviewer note: P6 had tried to give up… well, with smokes in her pocket!  ..*and at the time, I switched from Amber Leaf back to John Player and I went through nearly a pack a day and when I went onto the e-cigarettes, I went from a pack to a pack every five days, so I cut down a good bit yeah*. [Bradley, M, ESL]  Interviewe*r: And what do you think you would need to do to stop smoking?*  P4: *Pick up a hobby.*  P5: *Having more things to do like.*  P6: *Not being around people who are smoking.*  P2: *Yeah, not being around your friends, you’d have to lose your friends and all.*  P4: *Yeah, you’d have to stay in; you’d have to just talk to the wall.*  P2: *You couldn’t be with your friends.*  (a lot of inaudible conversation…)  Interviewer*: So, do a lot of your friends’ smoke? Like within your social circle?*  All: *Yeah.*  P2: *Everyone smokes.* [FG, F, ESL]  *…like they’re meant to get you off them* [pre-manufactured/ combustible cigarettes]. [Monica, F, ESL]  *The fact that they’re, they help you get off the smokes.* [Caitriona, F, ESL]  *You see, the way I started on vapes was I wanted to give up smoking and I tried the gums, I tried the sprays, I tried the patches and I tried cold turkey and they weren’t working like. You don’t get the same satisfaction. So, I said, I’ll try the vapes and they don’t work either, so I went back onto the rollies again. I haven’t smoked it* [e-cigarette] *for about a month*. [Simon, M, HES]  *It is like, I have tried to stop smoking over the years. Like I tried the e-cigarettes and then, I tried that for about a week and then went back on the smokes.* [Liffey, F, ESL]  Interviewer: *And how come you were continuing with the JP* (while using e-cigarettes)?  B: *Because I still wanted to smoke. I wouldn’t quit, I like smoking. I couldn’t imagine standing at a bus stop, waiting for a bus and not smoking, I wouldn’t be able for that.*[Bradley, M, ESL]  P4: *My ma has a pink one, she loves it, but she smokes as well.* [FG, F, ESL]  Interviewer: *Yeah yeah… Did you try it out of curiosity or because -*  C:  *Because I wanted to get off them like so I was like ‘Aw I’ll try that but …’ Yeah.* [Caitriona, F, ESL]  P6: *…I’m not even joking like, that electric smoke is not out of her hand. She’d light a smoke and then…*  Interviewer: *So, she would be using a cigarette and*…  P6: *Yeah like her smoke would be in her hand and then the other one is in the other hand. She’d be smoking the two of them at the same time. So, she’s actually using it, or like doubling it because she’s using an electric smoke, and she’s using the smoke. Like it’s not stopping her from smoking, because she’s smoking the same amount* [of cigarettes] *that she used to.* [FG, F, ESL]  *… a close friend of mine tried to wean her way off tobacco with them but I found she was constantly on it more so than if she was smoking tobacco, you know like, they’re constantly puffing it because you can constantly do it indoors as well. I think you’re more inclined to be doing it, she’s off the e-cigarettes and has just gone back to the rollies.* [Áine, F, HES]  *Me and my ma. She got one [e-cigarette] for herself and she got one for me, and said that we could quit together. My ma did it for about three months, I lasted about a week, I just couldn’t do it.* [Davis, F, ESL]  Interviewer: *OK right. And if you were going to quit would you think about using an e-cigarette?*  Ps: *No* (emphatic, consensus).  P3: *No, I’d just go cold turkey just to stop.*  Ps: *Yeah cold turkey* (consensus).  P1: *I’d rather get hypnotherapy or something like that (laughter). Like people say that if you get acupuncture and all it can help. I’d do all that crap before I’d go for an e-cigarette.*  P3: *And it can affect people differently too, like the different parts of whether you’re addicted to the habit of it or like the taste of it or like the break of it, y’know that kinda way.* [FG, F, HES]  P6: *I just… I think there’s no point like, if you’re gonna stop being addicted to something to be nearly addicted to something else, like I know you can get off them but I’d find like, if you’re going to be substituting a fag for an e-cig, it’s kind of… you might as well not…*  P4*: Yeah and I know so many people, like obviously not really my age, it’s more my parents’ friends and that kinda thing, that they would quit, go on an e-cig and then go back to smoking and then go back to the e-cig and it’s kinda a constant back and forth whereas if they kind of stopped, like they’re still going outside, standing out the back, doing that motion, it’s the same habit, so it’s… I think if I were to quit, obviously I’d cut down then just stop, that’d be my approach.* [FG, F, HES]  Interviewer: *And would many of your friends use e-cigarettes?*  N: *No, not many of them. A couple of them have tried them but most of them which have tried them just have ended up going back to smoking.* [Niamh, F, ESL]  Interviewer: *Were you just using it solely as a way to quit smoking was it?*  S: *Yeah*  Interviewer: *And what did you not find not helpful about it?*  S:  *You didn’t get the same satisfaction as having a smoke. It tasted different*  Interviewer: *Like not in a good way different?*  S: *No, like if you are looking for a smoke, you’re looking for the bad, the ashtray taste. You know it is synthetic and it just doesn’t feel the same so …* [Simon, M, HES]  Interviewer: *Yeah? And what about you guys? When you were smoking e-cigarettes, was that enough or did you want a real one as well?*  Participants: *A real one* (consensus). [FG, F, ESL]  D: *…* [I got the e-cigarette to stop smoking but] *it just left me more frustrated. I just felt that I had just a lack of a cigarette but also, that I was smoking this imitation that was nothing for me so it made me feel worse than not smoking at all.*  Interviewer: *And do you see it, the vape as a way to quit completely?*  D: *Yeah, I see it as a way to quit completely; at the moment, I’m using actual cigarettes as a crutch but I have a feeling that if I actually leave those aside, and just keep on the vape… the only thing is that you smoke more with a vape you know than you would with a cigarette*… [Daisy, F, HES]  Interviewer: Ok and it just didn’t work?  R: Yeah. I was just dying for a smoke and then I had the odd smoke still on the e-cigarette and then I just got rid of it (e-cig). [Liffey, F, ESL]  Monica – relapse  M: *but when I do see people around me smoking and I can just smell*  *it, I’m there puffing on that, I’d just wish that it was a real smoke in*  *my hand. Do you know that feeling but it did help me; I was on that*  *for a good six or seven months.*  Interviewer: *And just on that? You weren’t smoking?*  *M: No*  Interviewer: *And why did you give that up or stop using it?*  *M: I just stopped using that because it was too hard, I just needed to smoke.* [Monica, F, ESL]  Davis |
|  |  |
