# Supplementary material for: A qualitative study of e-cigarette use among young people in Ireland: Incentives, disincentives, and putative cessation
Source: PLoS One. 2020 Dec 28;15(12):e0244203. doi: 10.1371/journal.pone.0244203 (PMC7769428; doi:10.1371/journal.pone.0244203)
Supplement: S1 Table — (DOCX) [file pone.0244203.s004.docx]

S1 Table. Demographic, Smoking and E-cigarette use: Details of participants quoted**^*^**

| **Participant name**  **(pseudonym)** | **Gender** | **Education** | **Current**  **Smoker** | **Age first smoked**  **(in years)** | **Current E-cig user** | **Previous E-cig user** | **Current**  **dual User** |
| --- | --- | --- | --- | --- | --- | --- | --- |
| Áine | F | HES | Yes | 16 | No | No | No |
| Albert | M | ESL | Yes | 14 | No | Yes | No |
| Bradley | M | ESL | Yes | 13 | Yes | Yes | Yes |
| Caitriona | F | ESL | Yes | 12 | Yes | Yes | Yes |
| Daisy | F | HES | Yes | 17 | Yes | Yes | Yes |
| Damien | M | ESL | Yes | 8 | No | Yes | No |
| Davis | F | ESL | Yes | 13 | No | Yes | No |
| Finn | M | HES | Yes | 16 | No | No | No |
| John | M | HES | Yes | 16 | No | Yes | No |
| Liffey | F | ESL | Yes | 13 | No | Yes | No |
| Marnie | F | ESL | Yes | 14 | No | Yes | No |
| Monica | F | ESL | Yes | 12 | No | Yes | No |
| Niamh | F | ESL | Yes | 14 | No | Yes | No |
| Pascal | M | ESL | Yes | 16 | No | Yes | No |
| Paul | M | HES | Yes | 14 | No | Yes | No |
| Quinn | F | HES | Yes | 16 | No | Yes | No |
| Ron | M | ESL | Yes | 11 | No | Yes | No |
| Ryan | M | HES | Yes | 16 | Yes | Yes | Yes |
| Simon | M | HES | Yes | 16 | No | Yes | No |
| Tom | M | ESL | No | 12 | No | No | No |
|  |  |  |  |  |  |  |  |
| **FG M HES** |  |  |  |  |  |  |  |
| P1 | M | HES | Yes | NA | No | Yes | No |
| P2 | M | HES | Yes | NA | No | Yes | No |
| P3 | M | HES | Yes | 13 | No | Yes | No |
| P4 | M | HES | Yes | NA | No | Yes | No |
| P5 | M | HES | Yes | NA | No | Yes | No |
| P6 | M | HES | Yes | NA | Yes | Yes | Yes |
| P7 | M | HES | Yes | 15 | Yes | Yes | Yes |
|  |  |  |  |  |  |  |  |
| **FG F ESL** |  |  |  |  |  |  |  |
| P1 | F | ESL | Yes | 12 | No | Yes | No |
| P2 | F | ESL | Yes | 11 | Yes | Yes | Yes |
| P3 | F | ESL | Yes | 12 | No | Yes | No |
| P4 | F | ESL | Yes | 12 | No | Yes | No |
| P5 | F | ESL | Yes | 13 | No | Yes | No |
| P6 | F | ESL | No | 10 | No | Yes | No |
| **FG F HES** |  |  |  |  |  |  |  |
| P1 | F | HES | Yes | 14 | No | Yes | No |
| P2 | F | HES | Yes | 15 | No | Yes | No |
| P3 | F | HES | Yes | 20 | No | Yes | No |
| P4 | F | HES | Yes | 18 | No | Yes | No |
| P5 | F | HES | Yes | 15 | No | Yes | No |
| P6 | F | HES | Yes | 12 | No | Yes | No |
| **FG M ESL** |  |  |  |  |  |  |  |
| P1 | M | ESL | Yes | 13 | No | Yes | No |
| P2 | M | ESL | Yes | 12 | Yes | Yes | Yes |
| P3 | M | ESL | Yes | 14 | Yes | Yes | Yes |
| P4 | M | ESL | Yes | 13 | Yes | Yes | Yes |
| P5 | M | ESL | Yes | 8 | Yes | Yes | Yes |

**^*^** Interview and focus group participants whose quotes are used in MS or S3 Supplementary File only included in this Table.

**Age:** All participants aged 18-22 years.

**Legend:**

*Individual interviews*: Participant pseudonym; *Focus Group interviews*: P1 – P7 (Participant 1, Participant 2, etc.);

*All:* M/F (male/female); ESL (Youthreach= Early School Leaver) or HES (Higher Education Student); Current smoking status (yes/no); Age (in years) first smoked; Current e-cigarette user (yes/no); Previous e-cigarette user (yes/no – ‘yes’ includes current users); Current dual user (yes/no).
